# Effectiveness and Clinical Outcomes of PGT-M Using Karyomapping for Successful Pregnancy and Birth in Various Types of Charcot–Marie–Tooth Disease

**DOI:** 10.3390/jpm15070268

**Published:** 2025-06-23

**Authors:** Gaeul Han, Min Jee Kim, Ye Seul Hong, Shinhyung Lee, Jieun Lee, Ye Ryeong Lee, Hyoung-Song Lee, Kyung Ah Lee, Byung-Ok Choi, Eun Jeong Yu, Inn Soo Kang

**Affiliations:** 1 CHA Biotech, Seoul 04637, Republic of Korea; autumnmon92@gmail.com (G.H.); mjkim25@gmail.com (M.J.K.); hyeslh@gmail.com (Y.S.H.); shinh1031@chamc.co.kr (S.L.); jieun.lee@chamc.co.kr (J.L.); yrlee@chamc.co.kr (Y.R.L.); hslee999@gmail.com (H.-S.L.); 2 Department of Biomedical Science, College of Life Science, CHA University, Seongnam 13488, Republic of Korea; leeka@cha.ac.kr; 3 Department of Neurology, Samsung Medical Center, Sungkyunkwan University School of Medicine, Seoul 06351, Republic of Korea; bochoi77@hanmail.net; 4 CHA Fertility Center Seoul Station, CHA University School of Medicine, Seoul 04637, Republic of Korea; 5 CHA Fertility Center Daegu, CHA University School of Medicine, Daegu 41936, Republic of Korea; ikang67pgd@gmail.com

**Keywords:** Charcot–Marie–Tooth disease, preimplantation genetic testing for monogenic disorders, karyomapping, hereditary neuropathy

## Abstract

**Background:** Charcot–Marie–Tooth disease (CMT) is a genetically and clinically heterogeneous group of progressive peripheral neuropathies. Preimplantation genetic testing for monogenic disorders (PGT-M), a well-established assisted reproductive technology used to detect specific genetic mutations in embryos before implantation, has been used in common CMT subtypes (e.g., CMT1A); however, data on its application across rarer subtypes and in de novo cases remain limited. In this study, we aimed to evaluate the effectiveness of PGT-M using karyomapping in achieving clinical pregnancies and healthy births in families affected by various CMT types, including the previously unreported subtypes CMT1B and CMT2. **Methods:** We analyzed 31 PGT-M cycles from 13 families with genetically confirmed CMT, including cases of previously unreported subtypes CMT1B and CMT2. A total of 150 embryos were biopsied. Through 19 embryo transfer cycles, 21 embryos were transferred. In one de novo case, karyomapping was performed using amniotic fluid from an affected fetus as a reference. **Results:** Of the 19 embryo transfers, 15 resulted in clinical pregnancies. Prenatal diagnosis confirmed that all fetuses were unaffected, and all pregnancies resulted in healthy live births. Successful phasing using amniotic fluid from an affected fetus enabled accurate embryo selection and led to the birth of healthy twins. **Conclusions:** PGT-M using karyomapping is a rapid and reliable method for achieving successful pregnancies in families affected by diverse CMT subtypes, including de novo cases, and supports broader applicability to other monogenic disorders.

## 1. Introduction

Charcot–Marie–Tooth disease (CMT), also referred to as hereditary motor and sensory neuropathy, comprises a genetically and clinically heterogeneous group of progressive peripheral neuropathies [1]. CMT is characterized by distal muscle atrophy, muscular weakness, and sensory deficits [2], with a global prevalence estimated at approximately 1 in 2500 to 1 in 10,000 individuals [3]. Over 130 genes associated with CMT have been identified to date [4]. Owing to the extensive spectrum of genetic mutations, CMT is classified into several subtypes, typically grouped into three main categories: autosomal dominant neuropathies, including demyelinating (CMT1) and axonal (CMT2) forms; X-linked neuropathies (CMTX1); and autosomal recessive neuropathies [5]. Despite the genetic heterogeneity, up to 90% of genetically confirmed cases across cohorts are associated with mutations in four genes: *PMP22* duplication or deletion and mutations in *GJB1*, *MFN2*, and *MPZ* [6].

Preimplantation genetic testing for monogenic disorders (PGT-M) represents an assisted reproductive technology developed to detect pathogenic mutations in embryos prior to implantation [7]. Early applications of PGT-M involved direct mutation detection using single-cell PCR [8]. The development of multiplex fluorescence PCR enabled the simultaneous testing of relevant markers and mutations [8,9], allowing for haplotype identification even in CMT1A cases with *PMP22* alterations, including duplications or deletions [10]. However, conventional PCR approaches require approximately 6 months to design locus-specific probes [7].

To complement the conventional PCR-based approach for PGT-M diagnosis, karyomapping has been introduced as an alternative method [11]. Karyomapping provides a comprehensive, linkage-based diagnostic strategy for detecting any single-gene defect using over 300,000 single-nucleotide polymorphisms (SNPs) [12,13]. A key advantage of this method over traditional PCR-based techniques lies in its capacity to apply a unified protocol across a broad range of patients, thereby eliminating the need for patient-specific test development. Thus, this strategy offers the potential to substantially reduce patient waiting times [13].

We previously reported the first case of PGT-M using karyomapping for CMT1A, CMT2A, and CMT2S in Korea [14]. In this study, we report additional PGT-M cases using karyomapping for various CMT subtypes, including CMT1B, CMT2, and CMTX1.

## 2. Materials and Methods

### 2.1. Patient Characteristics

**Figure 1:**
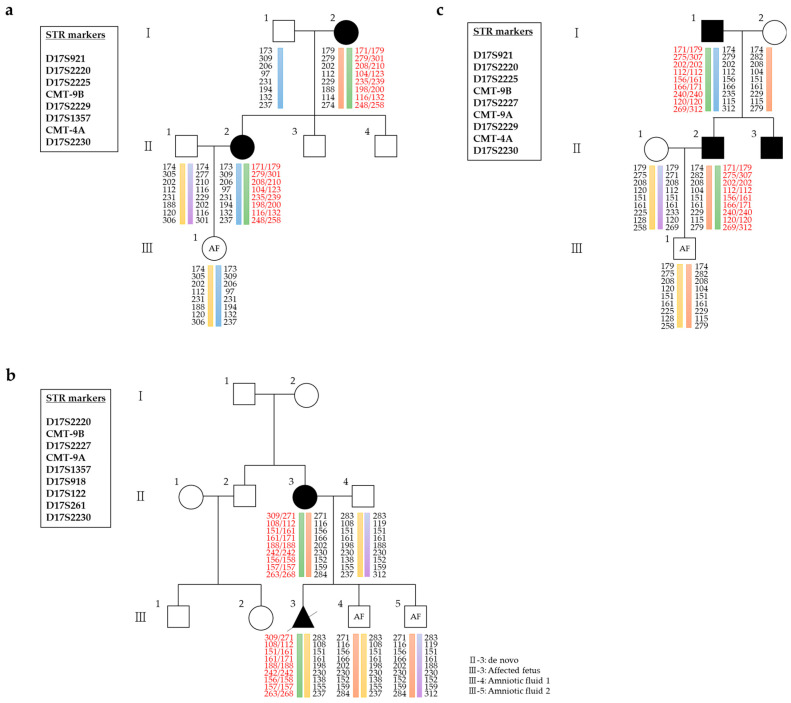
Pedigrees and haplotype analyses of three families with CMT1A, each carrying a 1.4 Mb duplication involving *PMP22*. The green bar indicates the mutant allele, while the other colored bars represent the normal allele. (**a**) Pedigree of Case 4 and the corresponding haplotype constructed using genotypes from 8 short tandem repeat (STR) markers. (**b**) Pedigree and haplotype of Case 5, based on genotyping of 9 STR markers. As this was a de novo case, the affected fetus—conceived naturally—was used as a reference for haplotype determination in subsequent karyomapping. (**c**) Pedigree and haplotype of Case 6, constructed using genotypes from 9 STR markers.

Thirteen patients diagnosed with CMT at various tertiary hospitals were referred to the Laboratory of Reproductive Genetics at CHA Biotech (Seoul, Republic of Korea) and underwent PGT-M testing. Between 2021 and 2023, 31 cycles of PGT-M using karyomapping were analyzed in 13 cases of CMT1, CMT2, and CMTX. PGT-M was requested for seven cases of CMT1A, two cases of CMT1B, one case of CMT2A, one case of CMT2, and two cases of CMTX1 (Table 1). Demographic and clinical information, including laboratory findings and clinical outcomes, were obtained from medical charts. Representative pedigrees for selected cases are shown in Figure 1.

Cases 1 to 7 were diagnosed with CMT1A, an autosomal dominant disorder caused by *PMP22* duplication. The male partner in Case 1 inherited the duplication from his mother, while in Case 2, the male partner inherited it from his affected father. Cases 3 and 4 involved affected male and female partners, respectively, who inherited *PMP22* duplication from affected mothers. Case 5 was a de novo female patient who conceived an affected baby naturally. Cases 6 and 7 involved affected male and female partners who inherited the *PMP22* duplication from affected fathers. Cases 8 and 9 were diagnosed with CMT1B, also an autosomal dominant disorder, characterized by mutations in *MPZ*. The male partner in Case 8 carried an *MPZ* c.410G>A mutation inherited from his father, and Case 9 involved a male partner with an *MPZ* c.242A>G mutation inherited from his mother. Case 10 involved CMT2A, an autosomal dominant disorder, where the male partner carried an *MFN2* c.839G>A mutation inherited from his affected father. Case 11 involved a female partner with an *MYH14* c.2822G>T mutation classified as CMT2 and inherited from her mother. Finally, Cases 12 and 13 had CMTX1, an X-linked dominant disorder caused by mutations in *GJB1*. The female partner in Case 12 carried a *GJB1* c.283G>A mutation inherited from her father, while in Case 13, the female partner carried a *GJB1* c.457T>G mutation inherited from her mother.

### 2.2. Preclinical Test for Karyomapping

All patients and families received genetic counseling, and informed consent was obtained for all procedures. Ethylenediaminetetraacetic acid (EDTA)-treated peripheral blood was collected from family members for DNA extraction using the QuickGene DNA Whole Blood Kit (Shimadzu, Neyagawa, Japan), according to the manufacturer’s protocol.

For the preclinical karyomapping test, DNA from affected family members was used. In one case, amniotic fluid from a previously affected fetus was used as the reference sample; in all other cases, the affected parent served as the reference. The use of residual prenatal samples was conducted under institutional ethical approval, with informed consent from the patient. This approach is consistent with current clinical guidance; the ASRM Practice Committee (2023) states that “For previous pregnancies, banked DNA samples (including those obtained from prenatal testing) may be useful” [15]. DNA from couples, reference family members, and amniotic fluid was analyzed using the HumanKaryomap-12 BeadChip according to the manufacturer’s protocol (Illumina, San Diego, CA, USA), and karyomapping was performed with BlueFuse Multi software version 4.5 (Illumina).

### 2.3. Embryo Biopsy and Whole-Genome Amplification

Following in vitro fertilization (IVF), embryos were cultured to the blastocyst stage and biopsied on day 5 or 6 using laser micromanipulation to extract approximately 5–7 trophectoderm (TE) cells. Blastocysts were morphologically graded per the Schoolcraft and Gardner guidelines [16]. Biopsied cells were washed in 3–4 microdroplets of phosphate-buffered saline (PBS) without magnesium or calcium and transferred to sterile PCR tubes containing 2 µL PBS. All embryos were vitrified and stored. Biopsied TE cells were processed for PGT-M. Whole-genome amplification (WGA) was performed using multiple displacement amplification according to the REPLI-g Single Cell Kit protocol (Qiagen, Hilden, Germany). WGA helped successfully generate sufficient genomic DNA for karyomapping analysis.

### 2.4. Karyomapping for PGT-M

Karyomapping was performed using the Infinium Human Karyomap-12 DNA analysis kit (Cat#1500055; Illumina). DNA fragmentation, precipitation, resuspension, hybridization, washing, and staining were performed per the manufacturer’s instructions. Data were scanned using the Illumina NextSeq 550 system and analyzed with BlueFuse Multi software (Illumina). Haploblocks were constructed based on genotypes from affected family members using informative SNPs from both partners. Mutant alleles within the haploblock were detected using genotype analysis. Mutant alleles were identified by analyzing heterozygous SNPs from trophectoderm cells within a 2 Mb region flanking the target gene.

### 2.5. Embryo Transfer and In Vitro Fertilization Outcome

Based on genetic counseling outcomes, a single unaffected blastocyst was thawed and transferred following endometrial preparation with artificial hormonal therapy. Clinical pregnancy was defined by the ultrasound-confirmed presence of a gestational sac and fetal heartbeat at 6–7 weeks of gestation.

### 2.6. Prenatal Diagnosis

Amniocentesis was performed under ultrasound guidance at 16–18 weeks of gestation, according to standard procedures [17]. DNA extracted from amniotic fluid cells was analyzed by (i) linkage analysis using STR markers as previously described [18], (ii) direct Sanger sequencing on a 3500 Genetic Analyzer (Applied Biosystems, USA) following the manufacturer’s protocol [19], (iii) multiplex ligation-dependent probe amplification with the P033 kit (MRC-Holland, Amsterdam, The Netherlands) according to the manufacturer’s instructions [20], (iv) chromosomal microarray using the CytoSNP-750K array (Illumina) on the GCS 3000Dx platform, and interpreted according to ACMG guidelines [21], (v) karyomapping as described by [11], and (vi) conventional G-band karyotyping, performed and interpreted using standard cytogenetic nomenclature.

## 3. Results

### 3.1. Preclinical Test Using DNA of the Affected Family

Genomic DNA from affected family members was used to identify informative SNPs linked to the mutant allele (Table 2). For CMT1A, the number of available SNPs in the 5′, main, and 3′ flanking regions of *PMP22* was 351, 15, and 233, respectively. In six of the seven cases, parental samples were used as the reference. However, in Case 5, the female partner was a de novo patient; therefore, an amniotic fluid sample from a previously affected pregnancy was used as the reference. For CMT1B, 152 and 193 SNPs were available in the 5′ and 3′ flanking regions of *MPZ*, respectively. In the case of CMT2A, 350, 6, and 200 SNPs were available in the 5′, main, and 3′ flanking regions of *MFN2*, respectively. For CMT2, involving *MYH14*, 96, 14, and 153 SNPs were available in the 5′, main, and 3′ flanking regions. Lastly, for CMTX1, 102 and 63 SNPs were available in the 5′ and 3′ flanking regions of *GJB1*, respectively.

### 3.2. Clinical PGT for CMT

Following preclinical testing, PGT-M with karyomapping was performed across 13 families affected by CMT (Table 3).

In DNA samples amplified using WGA, the mean SNP call rate was 91%, which was within the recommended range for blastocyst biopsy by Illumina (85–99%). The mean maternal age was 33.9 ± 3.1 years. Of the 150 embryos biopsied, 148 were successfully diagnosed (98.7%). Among these, 75 (50%) were identified as unaffected for CMT; of those, 23 were determined to be euploid and 27 were identified as having mosaicism. Twenty-five embryos with chromosomal abnormalities were excluded from embryo transfer. Across 31 biopsy cycles, 19 transfer cycles were performed, resulting in 21 embryo transfers and 15 clinical pregnancies (71.4%).

Linkage analysis, direct sequencing, and karyomapping were consistently used to diagnose embryos and validate outcomes through amniocentesis. Mosaic or aneuploid embryos were transferred only after comprehensive genetic counseling. Patients were fully informed about the potential reproductive risks and implications and gave written consent. All procedures were performed in accordance with institutional ethical guidelines.

#### 3.2.1. CMT1A

**Figure 2:**
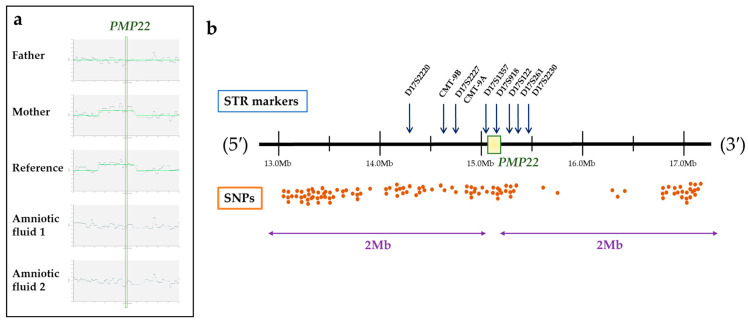
Log R ratio chart and marker/SNP distribution analysis in Case 5 with CMT1A. (**a**) Log R ratio charts for Case 5, demonstrating a duplication of the 1.4 Mb genomic region encompassing *PMP22* in both the CMT1A-affected mother and the affected fetus (reference sample), as compared to the unaffected father and the amniotic fluid sample, which exhibit normal copy number profiles. This duplication is consistent with the classical 1.4 Mb *PMP22* duplication typically observed in CMT1A. (**b**) Distribution of STR markers and SNPs across the *PMP22* 2 Mb flanking region, applied in the analysis of Case 5. The orange bins represent the distribution of informative SNPs, and the blue arrows indicate the positions of STR markers. Nine STR markers were informative in this region. In contrast, SNP genotyping identified a total of 116 informative SNPs: 73 located in the 5′ flanking region, 6 within the duplicated main region, and 37 in the 3′ flanking region. The large number of informative SNPs (116 in total) allowed for a more precise determination of the duplicated segment and its mode of inheritance.

Across seven CMT1A cases, 22 IVF cycles were performed, yielding 90 embryos. Among these, 41 were unaffected with respect to CMT, and 10 euploid or mosaic embryos were transferred. Eight pregnancies were achieved across the seven cases. Notably, in Case 5, where amniotic fluid from a previously affected fetus was used as the reference for karyomapping, a twin pregnancy was achieved following the transfer of unaffected embryos (Figure 2). All pregnancies resulted in the birth of healthy babies without complications.

#### 3.2.2. CMT1B

Two cases underwent two IVF cycles, resulting in 14 embryos, 9 of which were unaffected. Two embryos—one euploid and one mosaic—were transferred, leading to successful pregnancies and the birth of healthy babies in both cases.

#### 3.2.3. CMT2A

In Case 10, 11 embryos were obtained from one IVF cycle. Two transfer cycles resulted in two pregnancies. Karyotype analysis in both pregnancies revealed a pericentric inversion in chromosome 9 (p12q13), which is considered a benign variation. Both pregnancies resulted in healthy deliveries.

#### 3.2.4. CMT2

In Case 11, two IVF cycles yielded 12 embryos. A mosaic embryo with trisomy 21 was selected in the second transfer cycle, resulting in a successful pregnancy and the birth of a healthy baby.

#### 3.2.5. CMTX1

In two cases, four IVF cycles produced 23 embryos, 13 of which were unaffected. One embryo in Case 12 was successfully implanted, resulting in pregnancy. In Case 13, after two unsuccessful euploid transfers, a third cycle involved the transfer of one mosaic and one segmental aneuploid embryo, resulting in a singleton pregnancy. Karyomapping, karyotype analysis, and CMA confirmed a normal outcome. Both cases resulted in healthy births without complications.

## 4. Discussion

In this study, we demonstrate the clinical applicability and successful outcomes of PGT-M using karyomapping in various CMT subtypes.

Previously, PGT-M using PCR primarily focused on CMT1A, the most common subtype, and CMTX1, the second most frequent subtype [9,10]. Genome-wide karyomapping offers a significant advantage by being suitable for diagnosing any familial single-gene disorder [22,23], making karyomapping-based PGT-M a rational approach for identifying diverse CMT subtypes. Our center previously reported successful PGT-M cases using karyomapping for CMT1A, CMT2A, and CMT2S [14]. However, we had a small sample size (*n* = 4). In the current study, we expanded the cohort to 13 additional couples affected by CMT, enabling the identification of more subtypes (CMT1B, CMT2, and CMTX1). This increase addresses the earlier limitation and demonstrates the versatility of karyomapping. Notably, we present the first successful follow-up outcomes after PGT-M with karyomapping for CMT1B and CMT2.

SNP genotyping, which is used in karyomapping analysis, provides substantial advantages over conventional PCR-based diagnostics [24]. SNPs deliver comprehensive genetic information owing to higher genomic abundance (approximately one every 300–1000 bp) compared with that of STR markers [25,26]. The high SNP density enables precise identification of recombination events, which are challenging to detect using conventional PCR methods [13,24]. Additionally, karyomapping facilitates simultaneous analysis of preimplantation genetic testing for aneuploidy (PGT-A), as both SNP genotyping and chromosome copy number data are derived from raw sequencing data [26].

A limitation of karyomapping includes the requirement of a relevant family sample for phasing, potentially restricting applications for de novo mutations [26,27]. To overcome this, Konstantinidis et al. combined PCR with karyomapping using PCR-confirmed unaffected embryos as reference samples [13]. Alternatively, isolating a single sperm from an affected male provided a viable reference for paternally inherited mutations [13]. In all such cases, PCR and karyomapping results aligned consistently [13]. Additionally, we previously demonstrated that karyomapping accurately diagnoses embryos when using DNA extracted from tissue samples of a deceased affected sibling as the reference [28].

In Case 5 of our current cohort, involving a de novo patient, we used amniotic fluid from a previously affected fetus conceived naturally as the reference sample for karyomapping. This strategy facilitated a successful twin pregnancy and healthy births. These outcomes corroborate prior findings [28] and illustrate the potential of karyomapping to effectively overcome limitations in de novo patient scenarios.

While our findings demonstrate favorable clinical outcomes, it is important to acknowledge that trophectoderm biopsy—an essential step in PGT—may carry potential risks. Some studies have raised concerns regarding possible effects on implantation, embryo viability, and long-term child development. A recent review highlights the need for ongoing monitoring and ethical consideration in embryo biopsy protocols [29]. These factors underscore the importance of careful patient counseling and continued evaluation of the safety of such procedures.

In conclusion, we emphasize that clinical application of karyomapping-based PGT-M provides an effective, rapid alternative for achieving successful pregnancies and births in diverse CMT subtypes, including previously challenging de novo cases. These results further support the broader applicability of karyomapping-based PGT-M for families affected by single-gene disorders.

## Figures and Tables

**Table 1:** Genetic information of couples with CMT1A, CMT1B, CMT2A, CMT2, and CMTX1.

Couple	Affected Partner	Female’s Age (yr)	Phenotype	Inheritance Mode	Gene	Exon	Variant	Amino Acid Change	Reference Sequence Accession Numbers	Classification
1	Male	33	CMT1A	AD	*PMP22*	exon 1–5	17p11-p12 duplication	No amino acid change(duplication)	NM_000304.3	Pathogenic
2	Male	31	CMT1A	AD	*PMP22*	exon 1–5	17p11-p12 duplication	No amino acid change(duplication)	NM_000304.3	Pathogenic
3	Male	38	CMT1A	AD	*PMP22*	exon 1–5	17p11-p12 duplication	No amino acid change(duplication)	NM_000304.3	Pathogenic
4	Female	34	CMT1A	AD	*PMP22*	exon 1–5	17p11-p12 duplication	No amino acid change(duplication)	NM_000304.3	Pathogenic
5	Female	40	CMT1A	AD	*PMP22*	exon 1–5	17p11-p12 duplication	No amino acid change(duplication)	NM_000304.3	Pathogenic
6	Male	33	CMT1A	AD	*PMP22*	exon 1–5	17p11-p12 duplication	No amino acid change(duplication)	NM_000304.3	Pathogenic
7	Female	34	CMT1A	AD	*PMP22*	exon 1–5	17p11-p12 duplication	No amino acid change(duplication)	NM_000304.3	Pathogenic
8	Male	32	CMT1B	AD	*MPZ*	exon 3	c.410G>A	Gly137Asp	NM_000530.8	Pathogenic
9	Male	29	CMT1B	AD	*MPZ*	exon 3	c.242A>G	His81Arg	NM_000530.8	Pathogenic
10	Male	30	CMT2A	AD	*MFN2*	exon 9	c.839G>A	Arg280His	NM_014874.4	Pathogenic
11	Female	31	CMT2	AD	*MYH14*	exon 23	c.2822G>T	Arg941Leu	NM_001077186.2	Pathogenic
12	Female	33	CMTX1	XLD	*GJB1*	exon 2	c.283G>A	Val95Met	NM_000166.6	Pathogenic
13	Female	36	CMTX1	XLD	*GJB1*	exon 2	c.457T>G	Phe153Val	NM_000166.6	Likely pathogenic

CMT1A, Charcot–Marie–Tooth disease type 1A; CMT1B, CMT disease type 1B; CMT2A, CMT disease type 2A; CMT2, CMT disease type 2; CMTX1, CMT disease, X-linked dominant 1; *PMP22*, peripheral myelin protein 22; *MPZ*, myelin protein zero; *MFN2*, mitochondrial protein mitofusion-2; *MYH14*, myosin heavy chain 14; *GJB1*, gap junction protein beta 1.

**Table 2:** Informative SNPs identified using preclinical test cases with CMT1A, CMT1B, CMT2A, CMT2, and CMTX1.

	Case 1	Case 2	Case 3	Case 4	Case 5	Case 6	Case 7	Case 8	Case 9	Case 10	Case 11	Case 12	Case 13	
Type	CMT1A	CMT1A	CMT1A	CMT1A	CMT1A	CMT1A	CMT1A	CMT1B	CMT1B	CMT2A	CMT2	CMTX1	CMTX1
Affected partner	Male	Male	Male	Female	Female	Male	Female	Male	Male	Male	Female	Female	Female
Reference	Affected mother	Affected father	Affected mother	Affected mother	Amniotic fluid from the affected baby	Affected father	Affected father	Affected father	Affected mother	Affected father	Affected mother	Affected father	Affected mother
Target gene	*PMP22*	*PMP22*	*PMP22*	*PMP22*	*PMP22*	*PMP22*	*PMP22*	*MPZ*	*MPZ*	*MFN2*	*MYH14*	*GJB1*	*GJB1*
5′ region	20/351	25/351	27/351	21/351	73/351	28/351	18/351	11/152	15/152	44/350	10/96	43/102	17/102
Main region	1/15	0/15	0/15	0/15	6/15	0/15	0/15	0/0	0/0	0/6	2/14	0/0	0/0
3′ region	6/233	4/233	3/233	3/233	37/233	25/233	14/233	24/193	23/193	7/200	23/153	5/63	10/63

SNP, single-nucleotide polymorphism; CMT1A, Charcot–Marie–Tooth disease type 1A; CMT1B, CMT disease type 1B; CMT2A, CMT disease type 2A; CMT2, CMT disease type 2; CMTX1, CMT disease, X-linked dominant 1; *PMP22*, peripheral myelin protein 22; *MPZ*, myelin protein zero; *MFN2*, mitochondrial protein mitofusion-2; *MYH14*, myosin heavy chain 14; *GJB1*, gap junction protein beta 1.

**Table 3:** Clinical characteristics and outcomes of PGT-M using karyomapping across CMT subtypes, including CMT1A, CMT1B, CMT2A, CMT2, and CMTX1.

Clinical Data	Types					Total
	CMT1A	CMT1B	CMT2A	CMT2	CMTX1	
No. of couples treated	7	2	1	1	2	13
Maternal age (mean, years)	35.4 ± 2.9	31.0 ± 1.4	31	30	35.0 ± 1.4	33.9 ± 3.1
No. of OPU cycles performed	22	2	1	2	4	31
No. of oocytes retrieved	367	29	41	43	56	536
No. of oocytes fertilized (%)	211 (57.5)	21 (72.4)	30 (73.2)	28 (65.1)	42 (75.0)	332 (68.6)
No. of embryos biopsied (%)	90 (42.7)	14 (66.7)	11 (36.7)	12 (42.9)	23 (54.8)	150 (45.2)
No. of embryos diagnosed (%)	88 (97.8%)	14 (100%)	11 (100%)	12 (100%)	23 (100%)	148 (98.7)
Normal rate by CMT (%)	41 (45.6)	9 (64.3)	6 (54.5)	6 (50.0)	13 (56.5)	75 (50.0)
Abnormal rate by CMT (%)	47 (52.2)	5 (35.7)	5 (45.5)	6 (50.0)	10 (43.5)	73 (48.7)
No result (%)	2 (2.2)	0	0	0	0	2 (1.3)
No. of embryo transfer cycles	9	2	2	2	4	19
No. of embryos transferred (mean)	10 (1.1)	2 (1.0)	2 (1.0)	2 (1.0)	5 (1.2)	21 (1.1)
Clinical pregnancy rate per embryo transfer (%)	8/10 (80.0%)	2/2 (100%)	2/2 (100%)	1/2 (50.0%)	2/5 (40.0%)	15/21 (71.4%)
Miscarriage rate	0	0	0	0	0	0
Live birth rate	8/8 (100%)	2/2 (100%)	2/2 (100%)	1/1 (100%)	2/2 (100%)	15/15 (100%)

OPU, oocyte pick-up; CMT, Charcot-Marie-Tooth disease; CMT1A, Charcot-Marie-Tooth disease type 1A; CMT1B, CMT disease type 1B; CMT2A, CMT disease type 2A; CMT2, CMT disease type 2; CMTX1, CMT disease, X-linked dominant 1.

## Data Availability

The datasets generated and analyzed during the current study are not publicly available due to a concern to protect individual patient confidentiality but are available from the corresponding authors on reasonable request.

## Abbreviations

The following abbreviations are used in this manuscript:

CMT	Charcot–Marie–Tooth disease
PGT-M	Preimplantation genetic testing for monogenic disorders
SNPs	Single-nucleotide polymorphisms
EDTA	Ethylenediaminetetraacetic acid
IVF	In vitro fertilization
TE	Trophectoderm
WGA	Whole-genome amplification
PBS	Phosphate-buffered saline
CMA	Chromosomal microarray analysis
